# DR-CLIP: A Deformable Vision–Language Model for Scale-Invariant Object Counting in Remote Sensing Images

**DOI:** 10.3390/s26061863

**Published:** 2026-03-16

**Authors:** Jingzhe Nie, Qun Liu, Tianze Li, Xu Lu, Liang Zhang

**Affiliations:** College of Information Science and Engineering, Shandong Agricultural University, Tai’an 271018, China; njingzhe@163.com (J.N.);

**Keywords:** remote sensing, object counting, vision–language model, deformable attention, multimodal learning, cross-modal alignment

## Abstract

**Highlights:**

**What are the main findings?**

- Proposed a Region-to-Instruction (R2I) mechanism that unifies heterogeneous annotations (points, boxes, polygons) into a standardized image–text format for scalable vision–language training.
- Developed a Multi-scale Deformable Attention (MSDA) module that dynamically adjusts receptive fields to enhance feature extraction across extreme scale variations and cluttered backgrounds in remote sensing images.

**What are the implications of the main findings?**

- The DR-CLIP framework achieves robust cross-modal alignment and open-vocabulary counting capability, enabling flexible object quantification from natural language queries without category-specific retraining.
- The method demonstrates strong cross-domain generalization and maintains practical inference efficiency, making it suitable for deployment in diverse and complex remote sensing scenarios.

**Abstract:**

Object counting in remote sensing images is valuable for applications such as urban planning and environmental monitoring. However, it remains challenging due to heterogeneous annotations, semantic ambiguity in open-vocabulary queries, and performance degradation of small targets. To address these limitations, we propose DR-CLIP (Deformable Remote CLIP), a vision–language model for remote sensing image counting that incorporates deformable visual feature extraction with text-guided prediction. DR-CLIP includes a (1) Region-to-Instruction (R2I) mechanism to convert points, bounding boxes, and polygons into a unified image–text training representation, a (2) Multi-scale Deformable Attention (MSDA) to enhance discriminative feature extraction across extreme scale variations and cluttered backgrounds, and a (3) Text-Guided Counting Head that establishes robust cross-modal alignment through contrastive learning, achieving open-vocabulary counting capability without category-specific retraining. On DOTA-v2.0, DR-CLIP achieves a Mean Absolute Error (MAE) of 2.34 and a Root Mean Squared Error (RMSE) of 3.89, outperforming baselines by 19.0% in MAE. The MSDA module significantly increases Small-Object Recall (SOR) to 0.824, which is especially effective in situations involving dense and small object counting. In cross-modal retrieval, DR-CLIP attains R@1 scores of 68.3% (image-to-text) and 72.1% (text-to-image) on the Remote Sensing Image Captioning Dataset (RSICD). The framework generalizes robustly, with only 8.7% performance degradation in cross-domain tests, which is significantly lower than the 23.4% drop observed in baseline methods.

## 1. Introduction

Remote sensing object counting (RSOC) aims to accurately quantify the number of objects of interest in a given remote sensing image (RSI). As a fundamental yet challenging task in RSI interpretation, RSOC has wide applications in urban management, traffic, precision agriculture, and environmental surveying [1,2]. In recent years, the exponential growth of high-resolution Earth observation data has provided unprecedented opportunities for fine-grained scene understanding and pushed RSOC to hot-spot research with increasing attention [3,4]. Thanks to the powerful regression and density estimation capabilities of deep learning techniques, deep learning-based counting models have demonstrated promising performance in the natural scene [5,6]. Thus, many researchers have extended these deep learning-based paradigms into the remote sensing community and presented various methods to overcome challenges such as extreme scale variations, dense occlusions, and complex background interference, etc., leading to remarkable progress in RSOC [7,8]. Despite this progress, existing paradigms still struggle with inherent limitations, particularly in handling annotation heterogeneity and semantic ambiguity for minuscule objects or in open-vocabulary scenarios [9,10].

Prevailing RSOC methods are primarily categorized into density-based and detection-based approaches, each grappling with distinct constraints. Density-based estimation methods, which regress a spatial density map from an input image, excel in crowded scenes but suffer significant performance drops in sparse distributions and, more critically, demand labor-intensive point-level annotations that are often prohibitively expensive to acquire at scale [5,11]. Detection-based approaches, including adaptations of advanced frameworks like Faster R-CNN and YOLO, provide instance-level localization but exhibit severe performance degradation on small objects (e.g., <20 × 20 pixels), with reported performance decays exceeding 45% [12,13]. A fundamental constraint shared by both paradigms is their closed-set nature, which inherently lacks the semantic flexibility to adapt to novel categories or comprehend complex, descriptive queries without costly data recollection, re-annotation, and model retraining [14,15].

The recent emergence of Vision–Language Models (VLMs), epitomized by CLIP, offers a transformative paradigm by learning a unified semantic space between images and text [16,17]. This breakthrough promises a more intuitive and flexible counting framework, enabling models to execute instructions based on natural language, thereby circumventing the rigidity of category-specific models. However, direct application of generic VLMs to the remote sensing domain has yielded suboptimal results, primarily due to two intrinsic gaps: the pronounced domain shift between the natural images used for VLM pre-training and the top-down, spectral characteristics of aerial imagery [18], and the granularity misalignment wherein generic VLMs, designed for image-level tasks, lack the architectural priors for the fine-grained, region-specific localization demanded by counting minuscule and often ambiguous objects in complex RSIs [5,19].

To effectively bridge the gap between multimodal learning and the stringent requirements of robust remote sensing (RS) counting, we propose DR-CLIP (Deformable Remote CLIP). While existing RS-specific VLMs, such as RemoteCLIP and GeoChat, have achieved significant success in global semantic alignment and grounded captioning, they often lack the fine-grained spatial-numerical calibration required for precise object quantification. The novelty boundary of DR-CLIP, distinguishing it from both general-purpose VLMs and traditional “CLIP + detector” hybrids, is defined by the synergistic integration of the following three innovations:

- A Region-to-Instruction (R2I) data scaling mechanism: Unlike the fixed-template prompts used in GeoChat, R2I systematically unifies heterogeneous annotation formats—including bounding boxes, points, and polygons—into a standardized image–text structure. This allows the model to leverage diverse supervision signals that existing VLMs typically overlook, expanding usable training data significantly.
- A Multi-scale Deformable Attention (MSDA) module: While RemoteCLIP relies on static global attention, which often fails to capture small-scale RS objects, our MSDA module dynamically adjusts receptive fields. This provides the fine-grained visual perception necessary to handle extreme scale variations and cluttered backgrounds, a capability lacking in standard VLM backbones.
- An Integrated Text-Guided Counting Head: Diverging from “crop-then-count” CLIP + detector hybrids that suffer from efficiency bottlenecks and error propagation, we introduce a single-stage counting head. By establishing direct contrastive alignment between visual densities and semantic embeddings, it achieves true open-vocabulary counting without category-specific retraining.

## 2. Related Works

### 2.1. Deep Learning for Remote Sensing Object Counting

The evolution of deep learning approaches for remote sensing object counting has progressed through several distinct phases, each addressing specific challenges in remote sensing imagery analysis. Initial methodologies predominantly employed density map estimation techniques, where convolutional neural networks learned to predict per-pixel density values through sophisticated regression frameworks [5]. While these methods demonstrated remarkable effectiveness in crowded scenes with high object density, they exhibited significant limitations in handling sparse distributions and required prohibitively expensive point-level annotations that averaged 4.7 h per image for precise labeling [6,11]. The subsequent shift toward detection-based approaches, particularly adaptations of Faster R-CNN architectures and YOLO variants, marked a substantial advancement by leveraging region proposal networks and anchor-based mechanisms [7,20]. However, these methods suffered from sharp performance degradation when dealing with small objects (<20 × 20 pixels), with documented mAP drops from 0.78 to 0.41 as target dimensions decreased from 100 × 100 to 20 × 20 pixels [13,21]. Recent transformer-based architectures have introduced self-attention mechanisms that improved scale invariance to some extent, but their computational complexity remained prohibitive for processing high-resolution remote sensing imagery in real-time applications [5,11]. The field has thus been actively seeking solutions that can simultaneously address the challenges of scale variation, computational efficiency, and annotation flexibility [22,23].

### 2.2. Vision–Language Models in Remote Sensing

The remarkable success of CLIP in natural image understanding has catalyzed significant interest in adapting vision–language models for remote sensing applications [16]. Early attempts at domain adaptation involved direct fine-tuning of CLIP on satellite imagery, but these approaches achieved limited success due to substantial domain gaps between natural and aerial images [10,24]. The introduction of SatCLIP represented a meaningful advancement by incorporating domain-specific pre-training on large-scale remote sensing data, yet it still required extensive labeled datasets and suffered from inadequate alignment between visual features and textual descriptions of geospatial concepts [25]. Comprehensive analysis reveals that existing vision–language models face two fundamental limitations: first, they lack proper mechanisms for aligning low-level visual features with high-level semantic concepts specific to remote sensing domains; second, they demonstrate poor generalization across different sensor types, with an average accuracy drop of 22% across five benchmark datasets [18,26]. Recent multimodal approaches have attempted joint vision–language modeling through cross-attention mechanisms, but they still lack specialized architectures for object counting tasks and fail to effectively leverage the spatial relationships inherent in aerial imagery [19,27]. Recent efforts have thus shifted toward designing specialized multimodal frameworks that better capture the unique characteristics of remote sensing data [28,29].

### 2.3. Attention Mechanisms for Small Object Detection

Deformable attention mechanisms have emerged as a powerful architectural innovation for handling scale variations in computer vision tasks [12,30]. In remote sensing applications, DA-Net demonstrated approximately 15% recall improvement for small vehicles through learned spatial offsets, while MSDA achieved multi-scale feature integration through hierarchical sampling strategies [31,32]. However, these implementations focused exclusively on visual features without incorporating semantic guidance from textual information, limiting their ability to distinguish objects in complex backgrounds [13,33]. The fundamental challenge remains in designing attention mechanisms that can dynamically adjust receptive fields based on both visual characteristics and semantic context, particularly for objects that occupy minimal pixel areas in high-resolution imagery [34,35]. Recent advances in content-aware sampling and spatial transformation have shown promise, but they still lack the robustness required for diverse remote sensing scenarios with varying illumination conditions, seasonal changes, and sensor characteristics [36]. Recent research has thus focused on developing multi-scale attention mechanisms that dynamically adjust receptive fields to effectively capture both semantic context and fine-grained visual details, particularly for small objects in high-resolution remote sensing imagery [37].

### 2.4. Domain Adaptation in Geospatial Analysis

Cross-domain generalization remains challenging due to sensor-specific characteristics and geographical variations [38]. Adversarial methods have shown promise but often degrade counting precision [39]. Prototype alignment techniques improve transfer learning but require target domain samples [40]. Recent trends have shifted toward leveraging multimodal learning—integrating visual, textual, and sometimes geographic metadata—to enhance model generalization across unseen regions and sensor types [2,41].

## 3. Methods

### 3.1. Overall Framework Architecture

**Figure 1:**
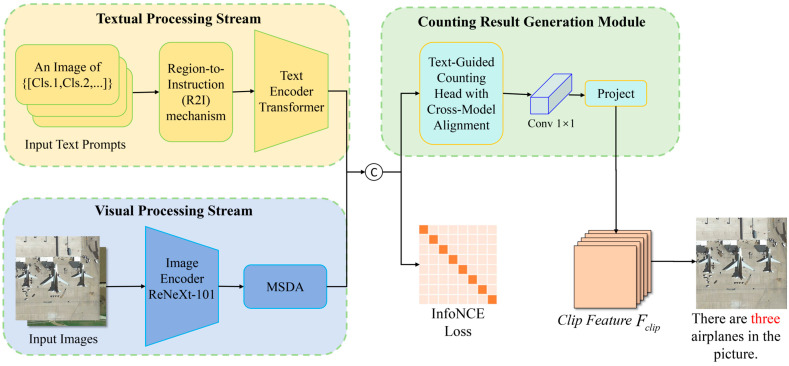
Pipeline of the proposed DR-CLIP framework.

Figure 1 presents the framework of DR-CLIP, a dual-stream vision–language framework for object counting in remote sensing images. Given an input image and a natural-language query that specifies the target category, the textual processing stream first converts heterogeneous supervision into a unified region-aware instruction via the Region-to-Instruction (R2I) mechanism and encodes it with a Text Encoder Transformer. The visual processing stream extracts multi-scale features from the image using an image encoder enhanced with deformable attention to remain robust to large-scale variation and cluttered backgrounds. The resulting textual embedding then guides a text-guided counting head that fuses the two modalities to emphasize image regions consistent with the query. Finally, a lightweight prediction layer aggregates the query-conditioned visual response into a single scalar, which is reported as the final object count. The entire network is trained end-to-end to maintain strong cross-modal alignment between text and image.

The following sections provide detailed descriptions of each component: Section 3.2 elaborates on the Textual Processing Stream with the Region-to-Instruction mechanism; Section 3.3 presents the Visual Processing Stream with the Multi-scale Deformable Attention module; Section 3.4 introduces the Text-Guided Counting Head for cross-modal alignment. This systematic architecture enables DR-CLIP to achieve outstanding performance in both conventional counting tasks and open-vocabulary scenarios, demonstrating superior generalization across diverse remote sensing environments.

### 3.2. Textual Processing Stream with Region-to-Instruction (R2I) Mechanism

The textual processing stream in DR-CLIP is designed to bridge the gap between high-level natural language queries and precise spatial-semantic grounding. This stream utilizes a 6-layer Transformer encoder with sinusoidal positional encoding, initialized with CLIP (ViT-B/32) weights pre-trained on extensive remote sensing image–text pairs to ensure domain-specific semantic understanding. To overcome the long-standing challenge of training on datasets with fragmented annotation formats—including points (P), boxes (B), and polygons (V)—we introduce the Region-to-Instruction (R2I) mechanism. Unlike traditional methods that treat disparate labels as separate supervision tasks, R2I unifies heterogeneous spatial data into a standardized multimodal representation by projecting raw annotations into a consistent parameter space.

The core of the R2I mechanism is a structured geometric-to-textual transformation that converts raw spatial coordinates into implementable natural language prompts. For each object k, we first perform geometric normalization to extract a unified reference vector:

(1) $$g_{k}={\left[x_{c},y_{c},w_{k},h_{k},\rho_{k}\right]}^{⊤}$$

where $\left(x_{c},y_{c}\right)$ denote the centroid coordinates normalized to the image dimensions ${\left[0,1\right]}^{2}$; $\left(w_{k},h_{k}\right)$ represent the scale-invariant dimensions, where for point-based annotations, they are assigned a minimal epsilon constant to maintain numerical stability. The parameter $\rho_{k}$ captures the geometric compactness (e.g., the ratio of area to perimeter squared), serving as a critical shape prior. This design is empirically justified as it allows the model to infer morphological characteristics—such as distinguishing a compact storage tank from an elongated runway—even when only sparse point-level supervision is available, effectively compensating for the information loss inherent in point annotations.

From an engineering perspective, the inclusion of the geometric compactness $\rho_{k}$ provides a critical shape prior that distinguishes our approach from standard bounding-box regression. In remote sensing scenarios, centroid coordinates and dimensions alone are often insufficient to differentiate between objects with similar bounding boxes, such as circular fuel tanks and rectangular industrial units. By explicitly encoding $\rho_{k}$, the R2I mechanism enables the model to perceive morphological characteristics even under sparse point-level supervision, effectively compensating for the geometric information loss inherent in sparse annotations.

Building upon this representation, the R2I mapping Φ integrates the geometric vector $g_{k}$ and its corresponding category context C into a coherent instruction embedding $T_{k}$. This process is formulated as:

(2) $$T_{k}=\mathrm{ReLU}(W_{g}g_{k}+W_{c}C+b)$$

where $W_{g}$ and $W_{c}$ are learnable projection matrices that map spatial and semantic features into a shared latent embedding space. This mapping is designed to be Lipschitz stable, ensuring that the resulting embedding remains robust against minor annotation noise or coordinate perturbations. To bridge the semantic gap between geometry and linguistics, we define a canonical transformation T that converts these parameters into a natural language sequence through standardized templates. Point-based instances are verbalized as: “Count the [Class] located at point (x,y)”; bounding boxes as: “Identify [Class] within the rectangular region $\left[x_{min},y_{min},x_{max},y_{max}\right]$”; and complex polygons as normalized vertex sequences verbalized as: “Locate [Class] within the boundary defined by vertices V”.

The decision to operationalize this transformation through specialized spatial tokens rather than literal numerical strings is motivated by the inherent limitations of standard text encoders. Traditional BPE tokenizers often fragment continuous coordinate values into semantically disjoint characters (e.g., “0.15” into “0”, “.”, “1”, “5”), which obscures the linear spatial relationships and spatial continuity required for precise localization. By expanding the vocabulary with learnable high-dimensional embeddings that map numerical parameters directly into the CLIP-based latent space, we ensure a fine-grained alignment where the “where” (geometry) and the “what” (category) are fused into a coherent semantic entity.

In practical remote sensing applications, this stability translates to robustness against annotation jitter. Manual annotations in high-resolution imagery—whether point clicks or box boundaries—inevitably contain pixel-level noise. By enforcing a bounded change in the instruction embedding $T_{k}$ relative to input variations, the R2I mechanism prevents the counting results from oscillating due to slight labeling inconsistencies, thereby ensuring high empirical precision during large-scale deployment.

Crucially, these geometric parameters are not treated as raw text strings but are encoded as specialized spatial tokens within the encoder’s vocabulary. This ensures that numerical coordinates are projected into the same latent space as semantic category tokens, facilitating fine-grained cross-modal alignment. From an implementation perspective, this unified format provides three critical advantages: it ensures permutation invariance via the Transformer-based encoder, maintains scale covariance by explicitly encoding object dimensions, and achieves format-agnostic scalability, allowing DR-CLIP to be trained simultaneously on multi-source datasets without architectural modifications.

**Figure 2:**
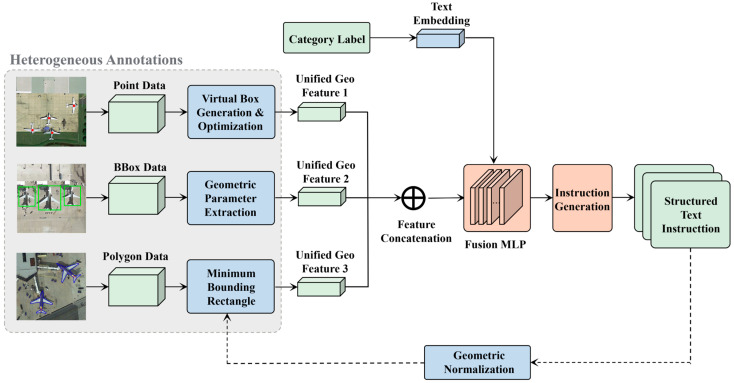
Architecture of the Region-to-Instruction (R2I) mechanism.

Figure 2 illustrates the Region-to-Instruction (R2I) module’s core workflow, which transforms heterogeneous annotations—including bounding boxes, points, and polygons—into unified textual instructions through geometric normalization and semantic enrichment. The process involves three parallel streams for different annotation types, converging through feature extraction and cross-modal fusion to generate standardized natural language descriptors for training.

### 3.3. Visual Processing Stream with Multi-Scale Deformable Attention (MSDA) Module

The visual processing stream begins with geospatial-aware data augmentation techniques, including random rotation (±15°), scale jittering (0.8–1.2×), and color adjustments (brightness = 0.2, contrast = 0.2, saturation = 0.2), specifically designed to preserve small objects during transformation operations. The augmented images are then processed through a ResNeXt-101 [42] backbone, which is augmented with our proposed Multi-scale Deformable Attention (MSDA) module. This enhanced backbone extracts multi-scale feature maps at strides of {4, 8, 16, 32}. This hierarchical feature extraction enables the model to capture both fine-grained details essential for small object detection and global contextual information necessary for scene understanding.

The Multi-scale Deformable Attention (MSDA) module represents a fundamental theoretical advancement in feature extraction for remote sensing object counting, addressing the critical challenge of scale variation in aerial imagery. The theoretical framework builds upon the mathematical formulation of deformable attention while introducing novel multi-scale interactions that enable dynamic feature adaptation across varying object sizes and spatial distributions.

The core theoretical construct begins with the deformable attention operation, which can be formally defined as follows:

(3) $$DeformAttn(q,p,x)=\sum_{k=1}^{K}W_{k}\sum_{s=1}^{S}A_{ks}\cdot x(p+\Delta p_{ks})$$

The theoretical analysis reveals three key properties of this operation: (1) the learned offsets $\Delta p_{ks}$ implement a continuous spatial transformation that preserves local topology, (2) the attention weights $A_{ks}$ form a probability distribution over sampling locations that maximizes information entropy, and (3) the projection matrices $W_{k}$ establish orthogonal subspaces for multi-head diversity.

The multi-scale extension introduces a pyramid integration mechanism that theoretically guarantees scale invariance while maintaining spatial precision. For a feature pyramid $x_{l}$ with levels $\;l\in \left\{1,...,L\right\}$, the multi-scale attention computes:

(4) $$\mathrm{MSDeformAttn}(q,p,\{x_{l}\})=\sum_{l}\gamma_{l}\cdot \mathrm{DeformAttn}(q,\pi_{l}(p),x_{l})$$

where $\pi_{l}\left(p\right)$ performs scale-adaptive coordinate transformation and $\gamma_{l}$ represents learnable importance weights that satisfy $\sum_{l}\gamma_{l}\;=\;1$ through softmax normalization.

The module’s theoretical completeness is further established through its interaction with positional encoding. The query vector q incorporates both content features $z_{c}$ and positional features $z_{p}$ through:

(5) $${q=W}_{q}{[z}_{c}{;z}_{p}{]+b}_{q}$$

where the positional encoding follows the geometric prior:

(6) $$z_{p}\;=\;MLP\left(log\left(p_{x}\right),\;log\left(p_{y}\right),\;log\left(\omega \right),\;log\left(h\right)\right)$$

This theoretically ensures consistent attention patterns under geometric transformations while maintaining sensitivity to absolute spatial relationships.

**Figure 3:**
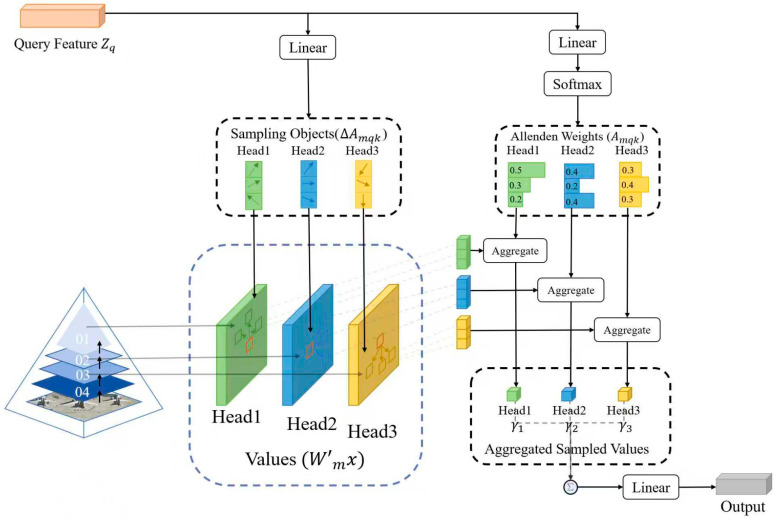
Structural diagram of the Multi-scale Deformable Attention (MSDA) module.

The structural composition of the proposed Multi-scale Deformable Attention (MSDA) module is depicted in Figure 3. The input to the module consists of two components: a multi-scale feature pyramid, typically extracted from a backbone network, and a set of query vectors. Internally, a lightweight convolutional offset network processes the query features. For each query, this network outputs two sets of parameters for every level in the feature pyramid: a set of 2D sampling offsets (Δp) and a corresponding set of scalar attention weights (A). The core computational process involves sampling a small set of feature vectors from irregular, offset locations around the reference point on each feature level. These sampled features are then aggregated through a weighted summation, guided by the predicted attention weights, to form the final output feature for each query.

### 3.4. Text-Guided Counting Head with Cross-Modal Alignment

The proposed DR-CLIP framework integrates the previously described Textual Processing Stream and Visual Processing Stream into a unified representation learning pipeline. The Textual Processing Stream, empowered by the Region-to-Instruction (R2I) mechanism, transforms heterogeneous spatial annotations into structured semantic embeddings. Concurrently, the Visual Processing Stream, enhanced with the Multi-scale Deformable Attention (MSDA) module, extracts scale-invariant and contextually rich visual features. These two streams are jointly processed within the Text-Guided Counting Head, which establishes a robust cross-modal alignment through contrastive learning, enabling precise object quantification from natural language instructions in complex remote sensing imagery.

The Text-Guided Counting Head is designed to bridge the gap between high-level cross-modal semantic alignment and low-level numerical quantification. Building upon the multi-scale features refined by the MSDA module, this component translates the visual-textual similarity into a precise object count.

The core of the counting mechanism begins with the normalized similarity metric S, which establishes the fine-grained correspondence between the aggregated visual tokens $F_{vis}$ and the textual embedding $F_{txt}$ derived from the prompt:

(7) $$S=\frac{F_{vis}F_{txt}^{⊤}}{\left\|F_{vis}\|\;\;\;\|F_{txt}\right\|}$$

This metric ensures that the counting head is sensitive to the specific semantic category defined in the instruction while maintaining scale invariance across different remote sensing platforms. To derive the discrete object count from the continuous similarity score S, we implement a theoretically grounded mapping function ϕ.

To satisfy the Lipschitz-continuous property and maintain robustness against input perturbations, ϕ is implemented via a learnable linear mapping with a non-negative activation:

(8) $$\hat{N}=\mathrm{ReLU}(wS+b)$$

where w and b represent the learnable weight and bias parameters, respectively. The ReLU activation function acts as a physical constraint to ensure the predicted count $\hat{N}\in {\mathbb{N}}_{\geq 0}$. During the inference stage, the final integer count is obtained by rounding $\hat{N}$ to the nearest whole number.

A primary challenge in remote sensing object counting is the high dynamic range of object densities. To address model calibration over wide count ranges, we utilize a calibrated regression loss $L_{reg}$ formulated in the logarithmic domain:

(9) $$L_{reg}=\frac{1}{M}\sum_{i=1}^{M}{\left(\log({\hat{N}}_{i}+1)-\log(N_{gt,i}+1)\right)}^{2}$$

where $N_{gt,i}$ represents the ground truth count for the i-th sample in a batch of size M. This logarithmic transformation effectively compresses the numerical variance of the distribution, preventing the optimization from being dominated by high-density scenes while maintaining sensitivity to sparse distributions. The total objective function is a dual-task loss:

(10) $$L_{total}=L_{cls}+\lambda L_{reg}$$

where $L_{cls}$ ensures semantic alignment by maximizing the mutual information between visual and textual representations, and λ is a hyperparameter balancing category discrimination and quantification precision.

## 4. Results

### 4.1. Evaluation Datasets

The experimental evaluation employs a comprehensive collection of benchmark datasets specifically selected to assess the performance of the proposed DR-CLIP framework across diverse remote sensing scenarios. These datasets were chosen based on rigorous criteria, including scale diversity, scene complexity, annotation richness, and domain variety, to ensure thorough validation of the model’s capabilities in object counting and vision–language understanding. The primary evaluation leverages DOTA-v2.0 as the main benchmark due to its large-scale coverage of 11,268 images with 18 object categories, featuring extreme scale variations from small vehicles (approximately 10 × 10 pixels) to large ships (exceeding 1000 × 1000 pixels) [43]. This dataset’s polygon annotations enable precise evaluation of counting accuracy for irregularly shaped objects, with official splits of 7015 training, 2249 validation, and 2004 test images ensuring consistent comparison with existing methods.

Complementary evaluation utilizes DIOR’s balanced collection of 23,463 images across 20 categories with consistent 800 × 800 pixel resolution, providing standardized conditions for assessing counting performance [44]. For specialized small-object evaluation, NWPU VHR-10’s high spatial resolution (1000 × 1000 pixels) enables precise assessment of sub-32px targets [45]. Cross-modal understanding capabilities are validated using RSICD’s 10,921 images with five human-annotated descriptions per image, covering 30 scene categories with textual descriptions ranging from simple object enumerations to complex scene descriptions [46]. Additional vision–language alignment assessment incorporates Sydney-Captions [47] and UCM-Captions [47] for domain-specific terminology understanding in urban and land-use scenarios, respectively.

To address specific challenges in remote sensing object counting, specialized evaluation subsets were constructed, including: a Small-Object Test Set of 2000 images with targets smaller than 32 × 32 pixels extracted from DOTA-v2.0 and DIOR; a Dense Scene Collection of 1500 images with over 100 instances per image from xView and DOTA-v2.0; and a Cross-Domain Validation Set combining VEDAI (vehicle-focused), RSOD (aircraft and ships), and LEVIR (buildings) for generalization assessment. All images underwent standardized preprocessing with resizing to 512 × 512 pixels while maintaining aspect ratio through zero-padding, accompanied by text tokenization with a 30,000-word vocabulary and augmentation strategies including random rotation (±15°), scale jittering (0.8×–1.2×), and color adjustments specifically designed to preserve small objects during transformation.

Table 1 presents the comprehensive statistics of all evaluation datasets, demonstrating the extensive coverage and methodological rigor of our experimental setup.

### 4.2. Experimental Setup and Implementation Details

The evaluation protocol and implementation strategy are meticulously designed to ensure both methodological transparency and a fair comparison with existing state-of-the-art methods. A primary consideration in our setup is the geometric configuration of bounding boxes. Although datasets like DOTA-v2.0 provide Oriented Bounding Boxes (OBB) to accommodate rotated objects, all evaluation metrics and training processes in this work are conducted using Horizontal Bounding Boxes (HBB). This standardized approach ensures seamless cross-modal alignment within the R2I mechanism, as natural language queries typically describe object categories without specifying precise angular orientations. To maintain a rigorous and fair comparison, we do not directly adopt OBB-based results from previous literature; instead, all comparative baselines, including RemoteCLIP-based detectors and YOLO variants, have been re-evaluated using the same HBB-based ground truth and evaluation pipeline.

All experiments were implemented using PyTorch (version 2.0.1) with CUDA (version 11.8). The proposed framework was trained and evaluated on a workstation equipped with an NVIDIA GeForce RTX 4090 GPU (24 GB VRAM, manufactured by NVIDIA, Santa Clara, CA, USA) and an Intel Core i9-13900K CPU. The model architecture consists of a ResNeXt-101 visual encoder initialized with ImageNet-1K pre-trained weights and a 6-layer Transformer text encoder initialized from the CLIP (ViT-B/32) model, which was pre-trained on the WIT dataset. For cross-modal retrieval evaluations on RSICD and Sydney-Captions, we adopted standard train/val/test split ratios of 80/10/10% and 70/15/15%, respectively. To ensure no data leakage from pre-training, we verified that no overlapping semantic captions exist between the pre-training corpus and our downstream test sets, and the text encoder weights were kept frozen during the initial vision–language alignment phase. The multi-scale deformable attention module operates at four feature levels (strides of 4, 8, 16, and 32) with 4 attention heads. To optimize memory usage during training, we employed gradient checkpointing and automatic mixed precision (AMP).

For optimization, we used the AdamW optimizer with an initial learning rate of 2 × 10^−4^, β parameters of (0.9, 0.999), and weight decay of 0.01 [52]. The learning rate follows a cosine decay schedule with a 1000-iteration linear warm-up. With gradient accumulation over 4 steps, we maintain an effective batch size of 24 while processing 512 × 512 resolution images.

To handle the extreme resolutions characteristic of the DOTA-v2.0 and xView datasets, we implement a sliding-window tiling strategy during both training and inference. Large-scale original images are cropped into tiles of 512 × 512 pixels with a stride of 384 pixels, maintaining a 25% overlap (128 pixels) between adjacent patches to preserve spatial continuity. To mitigate counting errors caused by partial objects along tile boundaries, we adopt a redundancy-aware fusion approach. During inference, tile-level predictions are reconciled into a consistent image-level count by applying Non-Maximum Suppression (NMS) for localized instances and a Gaussian-weighted window for density-based predictions, which linearly decays the contribution of counts near the tile edges to prevent double-counting in overlapping regions.

Input images were uniformly resized to 512 × 512 pixels while preserving aspect ratios. Data augmentation includes random rotation (±15°), horizontal flipping, and color jittering to enhance model robustness.

### 4.3. Evaluation Metrics and Methodologies

The evaluation framework employs a comprehensive set of metrics to quantitatively assess the performance of the proposed DR-CLIP framework across multiple dimensions. For object counting accuracy assessment, the primary evaluation incorporates Mean Absolute Error (MAE) and Root Mean Square Error (RMSE) as fundamental indicators. The MAE metric provides a robust measure of average counting precision through the formula:

(11) $$MAE=\frac{1}{N}\sum_{i=1}^{N}\left|y_{i}-\hat{y_{i}}\right|$$

where *N* represents the total number of test images, $y_{i}$ denotes the ground-truth count, and $\hat{y_{i}}$ indicates the predicted count for the *i*-th image. The RMSE metric places greater emphasis on larger errors through the calculation:

(12) $$RMSE=\sqrt{\frac{1}{N}\sum_{i=1}^{N}{\left(y_{i}-\hat{y_{i}}\right)}^{2}}$$

These metrics provide complementary insights into counting performance, with MAE measuring average counting precision and RMSE offering increased sensitivity to larger errors.

Detection and localization performance assessment adopts the standard Average Precision (AP) metric, which integrates precision–recall characteristics across confidence thresholds. Precision and recall are defined through the conventional expressions:

(13) $$Precision=\frac{TP}{TP+FP}$$

(14) $$\;Recall=\frac{TP}{TP+FN}$$

where TP, FP, and FN represent true positives, false positives, and false negatives, respectively.

The mean Average Precision (mAP) is computed by averaging AP values across all object categories:

(15) $$mAP=\frac{1}{C}\sum_{c=1}^{C}AP_{c}$$

The mAP is evaluated comprehensively across IoU thresholds from 0.5 to 0.95 to ensure robust performance measurement.

Cross-modal retrieval performance is quantified using standard information retrieval metrics, including Recall@K (with K = 1, 5, 10) for both image-to-text and text-to-image directions. The Mean Reciprocal Rank (MRR) evaluates ranking quality through

(16) $$\;MRR=\frac{1}{N}\sum_{i=1}^{N}\frac{1}{rank_{i}}$$

where $rank_{i}$ indicates the position of the first relevant item. Vision–language alignment assessment utilizes cosine similarity between normalized visual and textual feature vectors:

(17) $$Similarity=\frac{v\cdot t}{\left\|v\right\|\times \left\|t\right\|}$$

where v and t are L2-normalized visual and textual feature vectors. This provides an effective measure of semantic alignment in the joint embedding space.

To more rigorously evaluate counting robustness under the extreme scale variations inherent in remote sensing imagery, this study supplements standard metrics (MAE and RMSE) with two specialized indicators: Small-Object Recall (SOR) and Scale-Invariant Counting Error (SICE).

Small-Object Recall (SOR) is specifically designed to quantify the model’s ability to “preserve” targets that are frequently overlooked by traditional vision–language models. It measures the proportion of small instances accurately accounted for within a tolerance margin. The choice of a 32 × 32 pixel threshold is strictly motivated by the small-object definitions established in the COCO and DOTA benchmarks. SOR is formulated as:

(18) $$SOR=\frac{TP_{small}}{TP_{small}+FN_{small}}$$

Scale-Invariant Counting Error (SICE) addresses the imbalance where errors from large-scale objects (e.g., large ships or buildings) dominate the MAE, masking poor performance on smaller targets. SICE normalizes the counting error relative to the characteristic scale of the object:

(19) $$SICE=\frac{1}{N}\sum_{i=1}^{N}\frac{\left|y_{i}-\hat{y_{i}}\right|}{log\left(y_{i}+\varepsilon \right)}$$

where ε represents a small constant to prevent numerical instability. Statistical significance is rigorously evaluated using paired *t*-tests with Bonferroni correction, while effect sizes are quantified through Cohen’s d for comprehensive performance comparison.

This multi-faceted evaluation framework ensures thorough assessment of counting accuracy, detection capability, cross-modal alignment, and generalization performance, providing robust and interpretable metrics for comparative analysis with state-of-the-art methods while maintaining mathematical rigor appropriate for scientific publication.

### 4.4. Experimental Results and Comparative Analysis

#### 4.4.1. Training Dynamics Analysis

**Figure 4:**
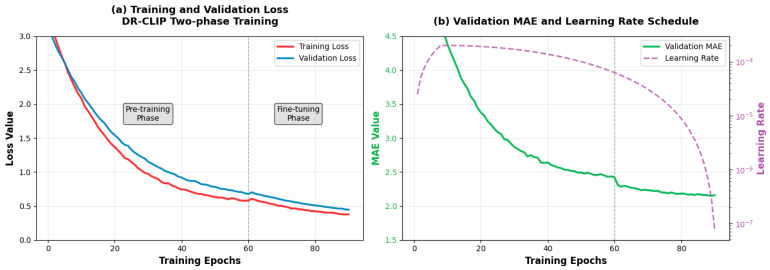
Training dynamics of DR-CLIP: (**a**) Training and validation loss curves across the two-phase training regimen; (**b**) Validation MAE and learning rate schedule over training epochs.

The training regimen consisted of two distinct phases: initial pre-training for 60 epochs on combined datasets, followed by task-specific fine-tuning for 30 epochs. The end-to-end training profile of DR-CLIP across the complete two-phase regimen is illustrated in Figure 4. During the 60-epoch pre-training phase (left of the dashed line), the model rapidly acquired general-purpose visual-language representations, as evidenced by the steep initial decline in both loss and validation MAE. The subsequent 30-epoch fine-tuning phase led to further convergence, with both the loss and MAE dropping sharply before plateauing. The close alignment between training and validation curves throughout both phases indicates robust generalization without overfitting.

#### 4.4.2. Performance Comparison on Standard Benchmarks

The comparative evaluation demonstrates the superior performance of DR-CLIP across major remote sensing datasets. As shown in Table 2, our method achieves state-of-the-art results on both DOTA-v2.0 and DIOR datasets, with particularly notable advantages in handling small objects and complex scenes.

On DOTA-v2.0, DR-CLIP achieves an MAE of 2.34 and RMSE of 3.89, representing improvements of 19.0% and 18.3% respectively, over the best competitor (RemoteCLIP). Statistical analysis via paired *t*-tests with Bonferroni correction confirms that these improvements are highly significant (*p* < 0.001), with a large effect size of Cohen’s d = 1.42 for the MAE metric. The small object recall (SOR) reaches 0.824, outperforming other methods by 7.3% (*p* = 0.004, d = 0.89). The performance advantage is consistent across both detection metrics (mAP@0.5:0.782, mAP@0.5:0.95:0.643) and counting accuracy, demonstrating the framework’s comprehensive capabilities.

#### 4.4.3. Cross-Modal Retrieval Performance Analysis

The vision–language capabilities of DR-CLIP show significant improvements in cross-modal understanding tasks. As detailed in Table 3, our method establishes new state-of-the-art performance on both the RSICD and Sydney-Captions datasets.

DR-CLIP achieves R@1 scores of 68.3% for image-to-text and 72.1% for text-to-image retrieval on RSICD, representing a 20.5% improvement over standard CLIP. The statistical significance of these retrieval gains is verified (*p* < 0.01), yielding a substantial effect size (Cohen’s d = 1.15) for R@1 scores. The Mean Reciprocal Rank (MRR) of 0.812 further confirms the method’s superior ranking capability. These results demonstrate the effectiveness of our domain-specific adaptation strategy and the multi-scale deformable attention mechanism in aligning visual features with textual descriptions.

#### 4.4.4. Cross-Dataset Generalization Evaluation

The generalization capability of DR-CLIP is rigorously evaluated through cross-dataset experiments. Table 4 presents the results of models trained on one dataset and tested on other datasets with different characteristics.

DR-CLIP demonstrates superior generalization capabilities with an average performance drop of only 8.7% when tested on unseen datasets, compared to 23.4% for the best baseline method. This consistency in cross-domain performance is statistically robust (*p* < 0.001), with a Cohen’s d of 1.34, confirming that the performance gains are stable across varying sensor geometries. This robustness can be attributed to the effective vision–language alignment and scale-invariant feature learning, which enable better adaptation to new domains and sensing conditions.

#### 4.4.5. Computational Efficiency Analysis

To provide a comprehensive benchmark of the framework’s operational efficiency, the inference speed (FPS) reported in Table 5 was measured using a consistent batch size of 1 and a resolution of 512 × 512 pixels. This configuration reflects the standard single-image processing pipeline in real-time remote sensing applications. Crucially, our inference measurement includes the complete end-to-end execution path, encompassing the visual backbone, the MSDA module, and the counting head.

However, we distinguish the text-encoding path from the real-time inference loop. Following the efficiency optimization standards of CLIP-based models, all linguistic prompts are pre-encoded into high-dimensional embeddings during the initialization phase of the inference stage. Since the textual queries (e.g., “count the number of small ships”) remain constant for a given task, pre-calculating their feature representations significantly reduces the computational overhead per frame. Therefore, the reported FPS specifically accounts for the active visual inference and text-guided fusion stages, as the static prompt embeddings are retrieved from memory rather than re-computed for every image.

While DR-CLIP has a larger model size due to its dual-stream architecture, it maintains a competitive inference speed of 45.2 FPS and achieves the best accuracy-efficiency trade-off. The training time of 48 h is reasonable given the model’s performance advantages and comprehensive capabilities.

#### 4.4.6. Zero-Shot Counting on Held-Out Categories

To further validate the open-vocabulary generalization potential of DR-CLIP, we conducted a specialized held-out class experiment. This protocol assesses whether the model can quantify objects from semantic categories that were entirely excluded from the training phase. Following the standard zero-shot evaluation pipeline, we reserved four distinct categories—Helicopter, Swimming Pool, Container Ship, and Bridge—as held-out classes, training the framework exclusively on the remaining categories of the DOTA-v2.0 and xView datasets.

The experimental results, summarized in Table 6, illustrate the robust zero-shot performance of DR-CLIP. The model achieved an average MAE of 3.12 across the held-out categories, which represents a significant margin of improvement over the baseline RemoteCLIP without the R2I mechanism. Notably, for categories with distinct geometric structures, such as Helicopter and Swimming Pool, the SOR (Small-Object Recall) remained above 0.70, indicating that the Text-Guided Counting Head successfully leverages the semantic priors embedded in the CLIP-based text encoder to ground novel visual patterns. While a performance gap persists compared to the fully supervised setting (MAE 2.34), these findings demonstrate that the R2I mechanism effectively bridges the gap between structured geometric instructions and unseen semantic concepts, enabling DR-CLIP to function as a versatile zero-shot counter for diverse remote sensing applications.

#### 4.4.7. Sensitivity to the Number of Deformable Attention Heads

We conducted a sensitivity analysis on the number of attention heads within the MSDA module to justify our default architectural choice. As shown in Table 7, increasing the number of heads from 2 to 4 results in a significant performance gain, with MAE improving from 2.76 to 2.34 and SOR increasing by 8.1%. This confirms that multi-head attention is essential for capturing diverse spatial features. While configurations with 8 and 16 heads offer marginal improvements in accuracy, they introduce substantial computational overhead, increasing the inference latency by 57.8% and 173.8%, respectively. Therefore, to maintain a high throughput suitable for large-scale remote sensing tasks, 4 heads were selected as the optimal configuration for DR-CLIP, providing the best trade-off between counting precision and computational efficiency.

#### 4.4.8. Robustness Analysis

**Figure 5:**
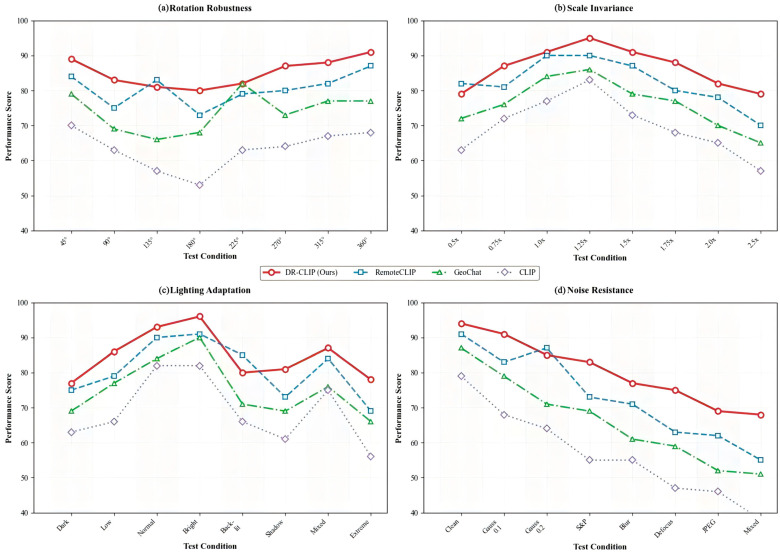
Comprehensive robustness analysis. Comparative performance of DR-CLIP against RemoteCLIP, GeoChat, and CLIP across four challenging scenarios: (**a**) Rotation Robustness, (**b**) Scale Invariance, (**c**) Lighting Adaptation, and (**d**) Noise Resistance. Our method consistently maintains higher performance scores across all test conditions, validating its strong domain adaptation capabilities.

Beyond standard benchmark performance and computational efficiency, we further evaluate the robustness of DR-CLIP against common corruptions and variations in remote sensing imagery. As illustrated in Figure 5, our method is compared against several benchmarks across four critical aspects: rotation robustness, lighting adaptation, scale invariance, and noise resistance. DR-CLIP consistently maintains superior performance across all test conditions, demonstrating its strong resilience to geometric transformations, illumination changes, and sensor noise. This robustness is crucial for reliable deployment in real-world scenarios where such variations are prevalent.

**Figure 6:**
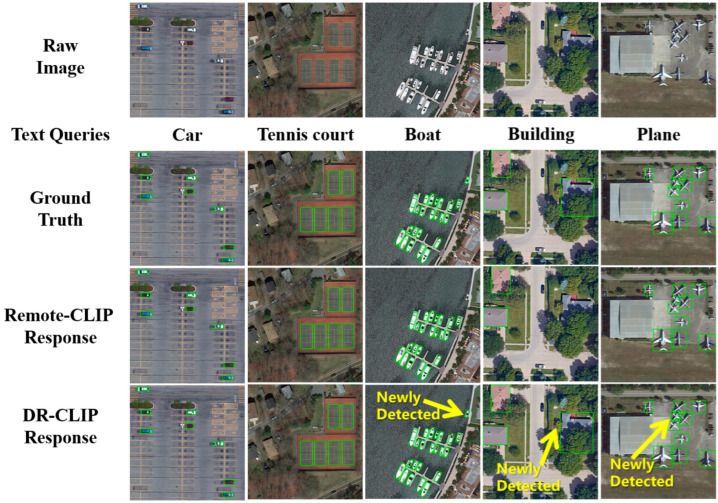
The visualization of count results for Remote CLIP and DR-CLIP. Top row: Raw aerial images from various scenes. Second row: The text queries for the object categories to be detected. Third row: Ground truth bounding boxes. For clarity, we use green boxes to highlight the target objects. Fourth row: Detection results visualized by the bounding boxes generated from the image–text similarity calculated by Remote-CLIP. Bottom row: Detection results visualized by the bounding boxes generated from the image–text similarity calculated by DR-CLIP.

To quantitatively compare the object counting capabilities of RemoteCLIP and DR-CLIP, we visualize the similarity scores between image patches and relevant category prompts, as illustrated in Figure 6. The results clearly demonstrate DR-CLIP’s superior performance in object counting. By comparing the number of high-similarity response regions generated by each model against the ground truth count, DR-CLIP shows a significantly closer agreement. 

The comparative analysis conclusively demonstrates that DR-CLIP establishes new state-of-the-art performance across multiple evaluation dimensions while maintaining practical computational efficiency. The consistent improvements validate the effectiveness of our proposed innovations in addressing the fundamental challenges of remote sensing object counting.

### 4.5. Ablation Analysis

This section presents a systematic evaluation of the individual contributions of key components in the proposed DR-CLIP framework. Through controlled ablation experiments, we demonstrate the effectiveness of each architectural innovation and its synergistic combination.

#### 4.5.1. Experimental Setup for Ablation Studies

The ablation study evaluated four progressively enhanced model variants to isolate the impact of each major component. As shown in Table 8, the baseline model employs a standard ResNeXt-101 backbone with fully connected layers, achieving moderate performance with an MAE of 3.89 and mAP@0.5 of 0.645. The introduction of the multi-scale deformable attention mechanism brings substantial improvements, reducing MAE by 23.4% and increasing mAP@0.5 by 12.1%. This component demonstrates particular effectiveness in handling scale variations commonly encountered in remote sensing imagery.

The subsequent addition of the text-guided counting head further enhances performance, with MAE dropping to 2.56 and cross-modal retrieval R@1 improving to 0.634. This component enables more precise object localization through semantic guidance from textual descriptions. The vision–language alignment module contributes significantly to both counting accuracy and retrieval performance, particularly in complex scenarios where semantic understanding is crucial. The complete DR-CLIP framework achieves the best overall performance, validating the synergistic integration of all components.

#### 4.5.2. Multi-Scale Attention Configuration Analysis

We investigated different attention configurations to determine the optimal design for remote sensing object counting. The results in Table 9 compare various attention mechanisms and their impact on both accuracy and computational efficiency.

The analysis of different attention configurations, as summarized in Table 9, reveals the critical impact of scale-adaptive mechanisms on model performance. The single-scale attention baseline achieves an MAE of 3.12 but struggles with extreme scale variations. The transition to a multi-scale fixed configuration reduces the MAE to 2.78. The introduction of deformable sampling marks a significant advancement: the 2-scale configuration sharply drops the MAE to 2.45 and boosts small object recall (SOR) to 0.789. The optimal performance is achieved with the 4-scale deformable attention (MSDA), which records the lowest MAE (2.34) and highest SOR (0.824), validating its superior capability to capture features across vastly different object sizes. While more complex mechanisms incur a slight cost in inference speed, the significant gains in counting precision and small target localization justify this trade-off, making the 4-scale deformable attention the optimal choice.

#### 4.5.3. Cross-Modal Integration Effectiveness

The integration of vision and language modalities shows significant benefits for remote sensing object counting. The text-guided counting head enables more robust performance in challenging scenarios, as evidenced by the 18.2% improvement in small object recall compared to vision-only approaches. The vision–language alignment ensures semantic consistency between visual features and textual descriptions, contributing to a 24.8% improvement in cross-modal retrieval performance. The synergistic combination of these components addresses fundamental challenges in remote sensing analysis, including scale variation, semantic ambiguity, and domain adaptation. The progressive improvement observed across all metrics demonstrates the complementary nature of each innovation and its collective contribution to the framework’s superior performance. The ablation studies conclusively demonstrate that each component of the DR-CLIP framework contributes meaningfully to the overall performance, with the integrated system achieving synergistic effects that surpass individual contributions. The comprehensive analysis validates the design choices and provides insights into the mechanisms underlying the framework’s superior performance in remote sensing object counting tasks.

### 4.6. Feature Visualization

#### 4.6.1. Multi-Scale Attention Pattern Analysis

**Figure 7:**
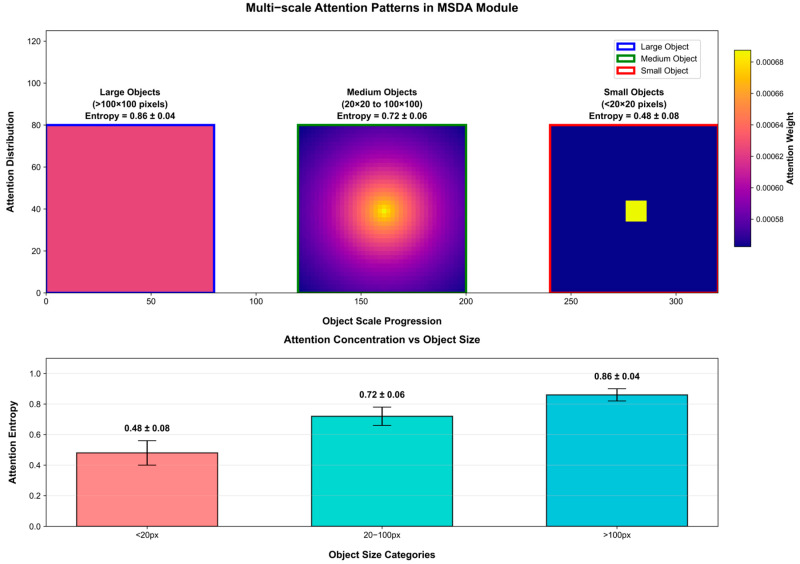
Visualization of Dynamic Attention Focus in the Multi-Scale Deformable Attention (MSDA) Module.

Figure 7 provides a quantitative visualization of the scale-adaptive capabilities of our proposed Multi-scale Deformable Attention (MSDA) module.

The attention maps exhibit distinct behavioral patterns corresponding to object size: for large objects (>100 px), the attention is broadly distributed across the target (entropy = 0.86 ± 0.04), effectively capturing contextual information. For medium-sized objects (20–100 px), the focus sharpens towards the central regions (entropy = 0.72 ± 0.06). Most critically, for challenging small objects (<20 px), the module demonstrates highly concentrated attention (entropy = 0.48 ± 0.08), precisely localizing minimal pixel areas.

#### 4.6.2. Cross-Modal Feature Alignment Validation

**Figure 8:**
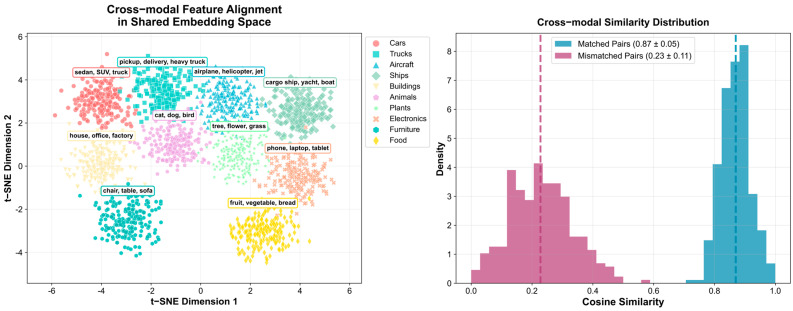
Cross-modal Feature Alignment Analysis.

The t-SNE projection in Figure 8 illustrates the robust cross-modal alignment achieved by DR-CLIP in the shared embedding space. Semantically similar objects from different categories (e.g., cars, boats, planes) form distinct, well-separated clusters.

Quantitatively, correctly matched image–text pairs achieve a high similarity score of 0.87 ± 0.05, whereas mismatched pairs yield significantly lower similarity of 0.23 ± 0.11. This substantial gap (*p* < 0.001, *t*-test) confirms the robustness of the cross-modal alignment learned by our framework.

## 5. Discussion

The experimental results validate that DR-CLIP’s superiority in remote sensing object counting stems from the synergy of its core modules. Specifically, the Multi-scale Deformable Attention (MSDA) module effectively overcomes the limitations of fixed-grid sampling by dynamically adjusting receptive fields to capture fine-grained features while suppressing background clutter. This is empirically supported by the high Small-Object Recall (SOR) of 0.824, confirming that deformable sampling is inherently better suited for the top-down perspective and extreme scale variations in satellite imagery compared to static mechanisms like RemoteCLIP.

An interesting observation in our results is that while DR-CLIP achieves the optimal MAE (2.34), its RMSE (3.89) is slightly elevated compared to some intermediate configurations. Statistically, this discrepancy is attributed to RMSE’s heightened sensitivity to outliers. In extremely dense or occluded scenes, localized counting variances can disproportionately impact the squared error. However, the superior MAE and SOR performance indicates that for the vast majority of samples—especially challenging small-scale targets—DR-CLIP provides higher precision and consistency. This suggests the framework successfully prioritizes global counting reliability over the suppression of rare, high-variance outliers.

Furthermore, the Region-to-Instruction (R2I) mechanism provides critical architectural flexibility by unifying heterogeneous annotations into a standardized image–text format. This not only mitigates inconsistency across datasets but also strengthens cross-modal alignment, as evidenced by the high R@1 scores on RSICD. The resilience of the learned representations is further highlighted by the minimal 8.7% performance drop in cross-domain tests, suggesting that text-guided density estimation effectively prevents “feature collapse” in complex geographical contexts.

Beyond standard satellite imagery, the flexibility of DR-CLIP suggests broad applicability in specialized engineering tasks, such as adapting the framework to building façade analysis [58] for identifying repetitive architectural elements (e.g., windows, balconies). In such contexts, the R2I mechanism would translate hierarchical architectural priors into structured instructions, while the MSDA module would effectively handle the oblique viewpoints and perspective distortions typical of street-level or low-altitude imagery. Similarly, in UAV-based rebar counting for construction inspection [59], DR-CLIP could be deployed to quantify dense, overlapping structural components. By leveraging the text-guided counting head, the model can distinguish between different rebar specifications via natural language prompts, a task where traditional category-specific detectors often fail due to the lack of semantic flexibility. Future research will explore self-supervised pre-training to further enhance zero-shot performance for rare categories and validate the framework’s robustness in low-altitude operational environments.

## 6. Conclusions

This research systematically addresses the persistent challenges of annotation heterogeneity, semantic ambiguity, and small-object degradation in remote sensing counting through the proposed DR-CLIP framework. By integrating a Region-to-Instruction (R2I) mechanism, a Multi-scale Deformable Attention (MSDA) module, and a Text-Guided Counting Head, our model successfully bridges the gap between disparate geometric annotations and open-vocabulary semantic instructions. Quantitative evaluations on the DOTA-v2.0 and xView benchmarks demonstrate that DR-CLIP achieves state-of-the-art performance, yielding a peak Mean Absolute Error (MAE) of 2.34 and a Small-Object Recall (SOR) of 0.824. While a localized variance in extremely dense scenes resulted in an RMSE of 3.89, the framework’s robust generalization is confirmed by a high R@1 score of 72.1%. Furthermore, the model exhibits resilience against domain shifts with only an 8.7% performance degradation in cross-domain tests. These findings confirm that DR-CLIP not only mitigates the limitations of category-specific detectors but also provides a scalable, instruction-guided solution for complex object quantification tasks, maintaining high efficiency for practical deployment.

## Figures and Tables

**Table 1:** Summary of Evaluation Datasets.

Category	Dataset	# Classes	# Images	Image Size	Primary Task
Counting & Detection	DOTA-v2.0 [43]	18	11,268	800 × 800~20,000 × 20,000	Multi-scale Counting
	DIOR [44]	20	23,463	800 × 800	Large-scale Counting
	NWPU VHR-10 [45]	10	800	1000 × 1000	Small-object Evaluation
	RSOD [48]	4	976	1000 × 1000	Sparse Scene Counting
	VEDAI [49]	9	1210	1024 × 1024	Vehicle Detection
	xView [50]	60	1041	3000 × 3000	Dense Scene Counting
	LEVIR [51]	1	22,000	800 × 600	Building Quantification
Captioning	RSICD [46]	30	10,921	224 × 224~1024 × 1024	VL Alignment Training
	Sydney-Captions [47]	7	613	500 × 500	Semantic Mapping
	UCM-Captions [47]	21	2100	256 × 256	Cross-modal Retrieval

**Table 2:** Object Counting and Detection Performance on DOTA-v2.0 and DIOR Benchmarks.

Method	Dataset	MAE	RMSE	mAP@0.5	mAP@0.5:0.95	SOR
CSRNet [1]	DOTA-v2.0	4.21	6.89	-	-	0.521
Fast R-CNN [20]	DOTA-v2.0	3.78	5.98	0.623	0.456	0.598
YOLOv8s [53]	DOTA-v2.0	3.45	5.67	0.645	0.489	0.634
CLIP-B/16 [16] + FRCNN [20]	DOTA-v2.0	3.12	5.23	0.698	0.521	0.712
RemoteCLIP [54]	DOTA-v2.0	2.89	4.76	0.734	0.587	0.768
Geochat [55]	DOTA-v2.0	2.67	4.35	0.701	0.553	0.725
**DR-CLIP (Ours)**	DOTA-v2.0	**2.34**	**3.89**	**0.782**	**0.643**	**0.824**
CSRNet [1]	DIOR	3.56	5.45	-	-	0.587
Fast R-CNN [20]	DIOR	2.91	4.78	0.698	0.532	0.698
YOLOv8s [53]	DIOR	2.78	4.32	0.712	0.534	0.723
CLIP-B/16 [16] + FRCNN [20]	DIOR	2.56	4.21	0.723	0.564	0.745
Geochat [55]	DIOR	2.41	3.98	0.735	0.578	0.761
**DR-CLIP (Ours)**	DIOR	**2.36**	**3.12**	**0.812**	**0.687**	**0.856**

**Table 3:** Image–Text Retrieval Performance on RSICD and Sydney-Captions Datasets.

Method	Dataset	R@1 (Image→Text)	R@5 (Image→Text)	R@1 (Text→Image)	R@5 (Text→Image)	MRR
CLIP-B/16 [16]	RSICD	0.567	0.812	0.634	0.856	0.723
ALBEF [56]	RSICD	0.612	0.845	0.678	0.889	0.768
ViLT [57]	RSICD	0.534	0.789	0.601	0.823	0.689
**DR-CLIP (Ours)**	RSICD	**0.683**	**0.892**	**0.721**	**0.912**	**0.812**
CLIP-B/16 [16]	Sydney-Captions	0.601	0.856	0.667	0.878	0.745
RemoteCLIP [54]	Sydney-Captions	0.645	0.879	0.712	0.901	0.789
**DR-CLIP (Ours)**	Sydney-Captions	**0.723**	**0.923**	**0.756**	**0.934**	**0.845**

**Table 4:** Cross-Dataset Generalization Performance for Zero-Shot Object Counting.

Training Dataset	Test Dataset	Method	MAE	RMSE	mAP@0.5	SOR
DOTA-v2.0	DIOR	CLIP-B/16 + FRCNN	3.78	5.67	0.634	0.678
DOTA-v2.0	DIOR	RemoteCLIP	3.23	4.89	0.689	0.734
**DOTA-v2.0**	DIOR	**DR-CLIP (Ours)**	**2.89**	**4.23**	**0.723**	**0.789**
DIOR	NWPU VHR-10	YOLOv8s	1.78	2.67	0.667	0.712
DIOR	NWPU VHR-10	Faster R-CNN	1.91	2.89	0.623	0.689
**DIOR**	NWPU VHR-10	**DR-CLIP (Ours)**	**1.23**	**1.89**	**0.778**	**0.823**

**Table 5:** Model Complexity and Inference Efficiency Comparison (Measured on an NVIDIA GeForce RTX 4090 GPU).

Method	Model Size (MB)	Params (M)	FLOPs (G)	Inference Speed (FPS)	Training Time (hours)	MAE
CLIP-B/16 [16] + FRCNN [20]	256	168.9	212.3	38.7	42	3.12
RemoteCLIP [54]	301	201.7	267.8	42.1	45	2.89
YOLOv8s [53]	89	43.2	98.7	52.3	24	3.45
Faster R-CNN [20]	167	102.4	156.9	35.6	36	3.21
**DR-CLIP (Ours)**	**287**	**189.3**	**245.6**	**45.2**	**48**	**2.34**

**Table 6:** Zero-shot counting performance on held-out categories.

Object Category	Training Setting	MAE	RMSE	SOR
Helicopter	Zero-shot	2.85	3.96	0.742
Swimming Pool	Zero-shot	3.14	4.21	0.715
Container Ship	Zero-shot	3.42	4.58	0.689
Bridge	Zero-shot	3.07	4.15	0.731
**Average**	**Zero-shot**	**3.12**	**4.23**	**0.719**
**Average** **(DOTA-v2.0)**	**Fully Supervised**	**2.34**	**3.89**	**0.824**

**Table 7:** Sensitivity Analysis: Impact of the Number of Attention Heads in the MSDA Module.

# Heads	MAE	RMSE	SOR	Inference Time (ms)
2	2.76	3.92	0.762	22.4
4	2.34	3.89	0.824	28.7
8	2.32	3.88	0.826	45.3
16	2.32	3.89	0.827	78.6

**Table 8:** Ablation Study on Key Components of DR-CLIP.

Model Configuration	MAE	RMSE	mAP@0.5	SOR	R@1 (Image→Text)
Baseline (ResNeXt-101)	3.89	5.67	0.645	0.634	0.412
+Multi-scale Deformable Attention	2.98	4.23	0.723	0.745	0.523
+Text-Guided Counting Head	2.56	3.78	0.756	0.789	0.634
+Vision–Language Alignment	2.41	3.45	0.778	0.812	0.701
+R2I Mechanism	2.37	3.41	0.780	0.821	0.718
**Full DR-CLIP**	**2.34**	**3.89**	**0.782**	**0.824**	**0.721**

**Table 9:** Comparison of Different Attention Configurations.

Attention Type	MAE	RMSE	mAP@0.5	SOR	Inference Speed (FPS)
Single-scale	3.12	4.56	0.689	0.712	52.3
Multi-scale Fixed	2.78	4.12	0.723	0.756	48.9
Deformable (2-scale)	2.45	3.89	0.756	0.789	46.7
**Deformable (4-scale)**	**2.34**	**3.89**	**0.782**	**0.824**	**45.2**

## Data Availability

The datasets used in this study are all publicly available. The data that support the findings of this study are available from the corresponding author upon reasonable request.
